# Hormonal influences on neuroimmune responses in the CNS of females

**DOI:** 10.3389/fnint.2013.00110

**Published:** 2014-01-17

**Authors:** Nela Monasterio, Edgar Vergara, Teresa Morales

**Affiliations:** ^1^Departamento de Neurobiología Celular y Molecular, Instituto de Neurobiología, Universidad Nacional Autónoma de MéxicoSantiago de Querétaro, México; ^2^Departamento de Biología, Facultad de Química, Universidad Nacional Autónoma de MéxicoCiudad de México, México

**Keywords:** stress, progesterone, prolactin, lactation, neuroimmune, HPA axis, female

## Abstract

Particular reproductive stages such as lactation impose demands on the female. To cope with these demands, her physiology goes through numerous adaptations, for example, attenuation of immune and stress responses. Hormonal fluctuation during lactation exerts a strong influence, inducing neuroplasticity in the hypothalamus and extrahypothalamic regions, and diminishing the stress and inflammatory responses. Thus, hormones confer decreased vulnerability to the female brain. This mini-review focuses on the adaptations of the immune and stress response during maternity, and on the neuroprotective actions of progesterone and prolactin and their effects on inflammation. The importance of pregnancy and lactation as experimental models to study immune responses and disease is also highlighted.

## INTRODUCTION

Reproduction is one of the most significant events in the life of a mammalian female. It can be described as a rich social and hormonal experience that begins by interacting and mating with a male, followed by pregnancy, parturition, and ultimately lactation, interaction with pups, and weaning (Mann and Bridges, 2001; Serafim and Felicio, 2002). Striking behavioral and neuroendocrine alterations due to motherhood have been reported in several mammalian species including mice, rats, rabbits, sheep, and humans. They are reflected by changes at almost all brain levels and are essential to protect the developing embryo, and for successful delivery, maternal behavior, and pup survival (Kinsley and Lambert, 2008). These neuroanatomical and functional changes observed during pregnancy and lactation are necessary to cope with the demands of reproduction and to protect the maternal organism against dramatic hormonal variations (Mann and Bridges, 2001; Kinsley and Lambert, 2008). They include a marked adaptation of the hypothalamus-pituitary-adrenal (HPA) axis, which results in hypo-responsiveness to stress, diminished inflammatory responses (Walker et al., 1992; Windle et al., 1997a; Lightman et al., 1998; Tilbrook and Clarke, 2006), and diminished sensitivity of the brain of the mother against excitotoxic damage (Morales, 2011).

## MECHANISMS GOVERNING THE HYPO-RESPONSIVENESS TO STRESS DURING PREGNANCY AND LACTATION

One of the best-known examples of naturally attenuated stress response is seen in the female rat during late pregnancy and lactation (Tilbrook and Clarke, 2006). During these reproductive phases, the HPA axis maintains minimal responses necessary to cope with any situation that may threaten the homeostasis of the female. This axis comprises corticotropin-releasing hormone (CRH)- and vasopressin- neurons in the paraventricular nucleus of the hypothalamus (PVN), which when stimulated, release these peptides into the median eminence to stimulate pituitary cell production of adrenocorticotropic hormone (ACTH) that reaches the adrenal cortex to release cortisol or corticosterone. HPA axis activity is regulated by glucocorticoid negative feedback on the pituitary, the PVN, and higher brain centers.

The attenuation of the stress response during pregnancy and lactation has been documented in various different stress models (Lightman et al., 2001; Russell et al., 2008). Pregnancy in the rat is accompanied by a progressive decrease in HPA axis responses to a range of psychological (Neumann et al., 1998) and physical (Brunton et al., 2005; Douglas et al., 2005) stressors particularly in the last week of pregnancy, reflected by reduced ACTH and corticosterone secretion. This hypo-responsiveness persists through parturition (Wigger et al., 1999; Neumann et al., 2003) and lactation until weaning (Windle et al., 1997b). Suppressed responses to stress in pregnancy can be explained by adaptations in both the anterior pituitary and the hypothalamus (Brunton et al., 2005; Russell et al., 2008). Corticotrophs in the pituitary are less sensitive to secretagogs (Shanks et al., 1997; Neumann et al., 1998), and CRH mRNA expression induced by stress is attenuated (Brunton et al., 2005). Moreover, HPA axis responses to immune stress in early mid pregnancy are strong and similar to that in virgins, although activation of hypothalamic vasopressin neurons, rather than CRH neurons, may be more important in the stress response in pregnancy (Ma et al., 2005; Parker et al., 2011).

Studies in lactating rats have shown a flattening of the diurnal rhythm of corticosterone secretion (Walker et al., 1992; Atkinson and Waddell, 1995) during this phase, such that the nadir levels of corticosterone rise (Stern et al., 1973; Fischer et al., 1995) and the peak evening levels decrease (Windle et al., 2013). During lactation, basal plasma concentrations of both ACTH and corticosterone increased in lactating animals compared with those in virgin rats (Shanks et al., 1997, 1999). There is also an increased basal expression of CRH mRNA in the PVN in early lactation (day 3; da Costa et al., 1996), but low basal expression of CRH mRNA by the middle phase of the lactation period (days 7–10; Windle et al., 1997a; Walker et al., 2001). However, CRH expression in response to stress is diminished at either stage (Lightman and Young, 1989; Windle et al., 1997b), similar to findings in late pregnancy (Douglas et al., 2005).

**FIGURE 1:**
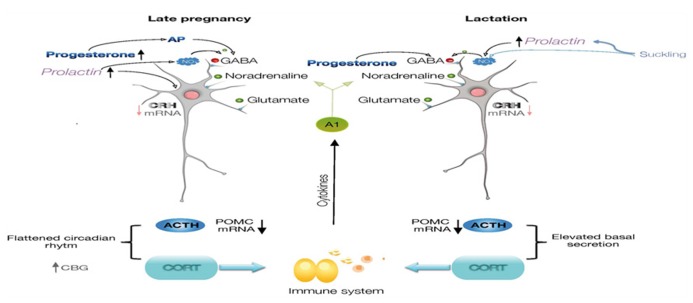
**Diagram illustrating the regulation of CRH expression in the parvocellular neurons of paraventricular nucleus of the hypothalamus.** During late pregnancy and lactation, there is a diminished noradrenergic tone reaching CRH neurons, and progesterone, allopregnanolone, prolactin, and nitric oxide will contribute to the inhibitory regulation of the CRH neurons. The cascade of neuroendocrine responses, synthesis of ACTH and corticosteroids will also be modified. Neuroimmune interactions are represented by effects of glucocorticoids on cells of the immune system, and which molecules will affect CNS functions. ACTH, adrenocorticotropic hormone; AP, allopregnanolone; CRH, corticotropin-releasing hormone; CBG, corticosteroid-binding globulin; POMC, proopiomelanocortin.

The mechanisms for this altered neuroendocrine responses include diminished CNS excitatory signaling within stress-responsive systems like the catecholaminergic brainstem and limbic circuitry (Hoffman et al., 1994; da Costa et al., 1996; Wintrip et al., 1997) as well as an altered hypothalamic and adrenomedullary catecholamine function (Patel et al., 1993; Toufexis and Walker, 1996; Windle et al., 1997a; Toufexis et al., 1998). Attenuated noradrenergic tone also underlies stress hypo-responsiveness in lactation, and is clearly dependent upon suckling (Hoffman et al., 1994; Toufexis and Walker, 1996; Toufexis et al., 1998). Moreover, opioids switch to having a net inhibitory effect on HPA activity such that naloxone treatment enhances the ACTH response to IL-1β in late pregnant rats (Brunton et al., 2009). Growing evidence for an integrated participation of many other factors besides noradrenaline and opioids that underlie altered responses to stress, include CRH (Johnstone et al., 2000; da Costa et al., 2001), oxytocin (Neumann et al., 2003; Windle et al., 2004), prolactin (PRL; Torner and Neumann, 2002), steroids and neurosteroids (Douglas et al., 2000; Brunton and Russell, 2008, 2011; **Figure 1**).

Another mechanism for the attenuation of the stress response involves nitric oxide (NO) regulation of CRH expression within the PVN. NO is present in the PVN of female rats during lactation (Popeski et al., 1999; Otukonyong et al., 2000; Monasterio and Morales, 2011), as indicated by the NO markers NADPH-diaphorase and neuronal synthase (nNOS), and its synthesis dependent on suckling (Otukonyong et al., 2000) and PRL (Vega et al., 2010). These markers increase within the PVN in response to stress in male rats, but paradoxically, basal level of NO markers are present in the PVN of lactating females whose response to stress is attenuated. The activational c-fos response and an increase in NADPH-diaphorase- and nNOS-positive cells are clearly detected in the PVN of diestrus rats after an immune challenge, but not in lactating rats. Furthermore, the total concentration of nitrates in the hypothalamus and the circulating level of corticosterone and IL-6 increase significantly after stress in diestrus, but not in lactating rats, compared to their corresponding controls. Intracerebral administration of L-NAME, a general NOS inhibitor, reverses the attenuation of the activational response to stress in the lactating rats and increases CRH expression, suggesting that sustained NO production in the PVN during lactation contributes to attenuate the neuroendocrine response to stress (Monasterio and Morales, 2011; **Figure 1**).

## HPA AXIS HORMONES, PROGESTERONE, AND PROLACTIN IN THE REGULATION OF THE IMMUNE RESPONSES DURING PREGNANCY AND LACTATION

### HPA AXIS AND IMMUNE RESPONSES

Cytokines, peptide hormones, neurotransmitters, and their receptors are endogenous elements of the nervous, endocrine, and immune systems. These systems share ligands and receptors that serve to communicate (Haddad et al., 2002; Elenkov and Chrousos, 2006). In addition to altered endocrine responses during lactation, stress also changes immune function in comparison to non-lactating animals (Monasterio and Morales, 2011), suggesting that bi-directional communication between the immune and endocrine systems is also altered during lactation. Pregnancy and lactation represent interesting experimental models that illustrate how the elevated basal plasma level of corticosterone and the diminished stress responses, affect the immune system. It is established that dampened HPA responses are associated with increased vulnerability to inflammation (Shanks et al., 1997, 1999) and therefore, alterations in endocrine regulation of immune responses during lactation may predispose the animal to inflammatory disease. However, not all of the immune responses are dampened during pregnancy and lactation (Jaedicke et al., 2009).

Several reports have documented motherhood-induced adaptations of the immune system in female rats, showing that this is a not always an immune suppression condition (Jaedicke et al., 2009). Early pregnancy has been associated with an increased cell density in the thymus and spleen, whereas late pregnancy and lactation have been associated with a decreased cellularity despite the expansion of the thymus medulla (Kendall and Clarke, 2000). Lactation delays the return of the thymus medulla to the original pre-pregnancy state. In addition, the changes in the thymus can be attributed to the adaptation of the maternal immune system to the semi-allogeneic fetus (Kendall and Clarke, 2000). Hormonal and neural changes can partially explain these modifications. Progesterone, PRL, and estradiol levels in plasma fluctuate in early, mid, and late pregnancy (Neville et al., 2002; Brusco et al., 2008). Nerve growth factor, nearly absent in the medulla of the adult thymus, is high in the medulla of the thymus during late pregnancy (Aloe et al., 1997). Also, lactation is associated with an increased susceptibility to parasitic infections (Barger, 1993), but much controversy exists in the effects of pregnancy and lactation on the progression of autoimmune diseases as documented by studies in rodents and in humans (Elenkov et al., 2001; Buchel et al., 2002; Vukusic et al., 2004; Gregg, 2009).

The immune system of lactating rodents has been the focus of only a few studies, which have shown that some immune functions become suppressed in this phase, while others remain unaffected or are enhanced (Jaedicke et al., 2009). For example, antibody production after immunization, and IL-6 (Monasterio and Morales, 2011) and IL-2 production in the spleen (Shanks et al., 1997) were suppressed during lactation in rodents. Conversely, evidence of increased concentrations of plasma IL-6 or an enhanced proliferative response of lymphocytes from mesenteric lymph nodes suggests activation of other immune responses (Shanks et al., 1997). In male rats, activation of the HPA axis and glucocorticoid release occurs during bacterial lipopolysaccharide (LPS) exposure, triggering an elaborate inflammatory response that involves the release of the pro-inflammatory mediators IL-1β, IL-6, and tumor necrosis factor (TNF)-α (Grinevich et al., 2001), which stimulate cells to upregulate the inflammatory reaction, and systemically they activate the HPA axis (Chrousos, 1995). However, in lactating rats, LPS significantly elevates circulating levels of ACTH and corticosterone, but the magnitude of hormonal and hypothalamic responses to LPS are significantly reduced in lactating animals relative to virgin controls (Shanks et al., 1999). Despite this attenuated stress response, systemic immune responses to stressors are modulated during lactation indicating that the immune system is not generally suppressed but rather adjusted in this stage (Jaedicke et al., 2009).

In humans, the early postpartum period has been associated with up-regulated inflammatory responses and a relapse of autoimmune disorders such as rheumatoid arthritis and multiple sclerosis, often interpreted as a flare-up due to the rebound of the immune system after pregnancy (Elenkov et al., 2001; Buchel et al., 2002). However, long term-studies have shown that relapse rate of multiple sclerosis remains similar to pre-pregnancy level, after an increase in the first trimester postpartum (Vukusic et al., 2004). A broad state of immune activation is also characteristic of the early postpartum period, as measured by levels of neopterin, soluble IL-2 receptor, and soluble CD8 antigen (Burns et al., 1999). This may help women recover from the biological stress of parturition, but more studies about the magnitude and length of such a state are necessary. Immune and inflammatory activation in postpartum women may be factors leading to anxiety and depression in the early days after delivery (Maes et al., 2000). CD4 cell counts are reported to rise during postpartum, primarily due to γδ T cells (Watanabe et al., 1996). Natural killer (NK) subsets with weak cytotoxic activity (CD16+, CD57+) were found to increase during months 1–4 postpartum (Watanabe et al., 1997), and lymphocyte proliferation was higher than non-postpartum controls. Furthermore, the postpartum period is also associated with the onset of autoimmune thyroid syndrome (Muller et al., 2001). Thus, immune responses linked to reproduction can either be dampened or enhanced depending on the stimulus and hormonal status (De Bellis et al., 2005; Hughes, 2012; Shelly et al., 2012).

In summary, during both pregnancy and lactation the immune responses are altered, but instead of considering them to be immune-suppressed stages, they represent reproductive conditions in which either stress or immune responses can vary depending on the hormonal status (Mor and Cardenas, 2010). The immunology of reproduction is the result of the combination of signals and responses originating from the fetal-placental and the maternal immune systems. This last is under the regulation of the CNS through the hormonal (glucocorticoids) stress response, pituitary responses, and the autonomic nervous system.

### PROGESTERONE ACTIONS ON CNS IMMUNE RESPONSES

Among the hormones that have been considered candidate inducers of pregnancy- and lactation-related adaptations in HPA axis and immune responses is progesterone. Progesterone is a steroid hormone synthesized by the corpus luteum in cycling females and by the placenta during pregnancy; it can enter the brain from the circulation and can also be synthesized in the brain by oligodendrocytes and excitatory neurons (Stein, 2011). During pregnancy, progesterone levels in plasma and brain are increased and are accompanied by elevated levels of its metabolite allopregnanolone (Brunton and Russell, 2008; Mostallino et al., 2009).

Progesterone (and its metabolite allopregnanolone) has important actions in the female’s brain, such as the expression of maternal behavior (Bridges, 1984). During pregnancy, high levels of allopregnanolone suppress HPA -axis responses to stress: allopregnanolone may enhance the action of GABA in the PVN or on afferent inputs to the CRH neurons to suppress stress responses, and it also induces and maintains the endogenous inhibitory opioid mechanism in the nucleus of the solitary tract (Brunton et al., 2009). Blocking allopregnanolone restores HPA axis responses to systemically administered IL-1β in late pregnant rats (Brunton and Russell, 2008). Allopregnanolone acts as an agonist on the GABA receptor, exerting anxiolytic, sedative, and antiepileptic effects, and it enhances the myelination/remyelination process in the central and peripheral nervous system (Wang et al., 2008; Gangisetty and Reddy, 2010). These steroid hormones have been described as potent regulators of growth factor expression during pregnancy: epidermal growth factor (EGF), insulin-like growth factor (IGF), and transforming growth factor (TGF-β1) in particular are all up-regulated, promoting neural proliferation (Wang et al., 2008). Moreover, they increase neurogenesis within the subgranular zone of the dentate gyrus and subventricular zone (Pawluski et al., 2009), and induce regenerative responses in a mouse model of Alzheimer’s disease (Pike et al., 2009; Borowicz et al., 2011). During lactation, progesterone participates in glial changes in brain areas such as the cingulate cortex (Salmaso et al., 2009) and the dentate gyrus of the hippocampus (Cabrera et al., 2013), and this steroid is part of the hormonal cocktail responsible for diminished responses of astrocyte and microglial cells in the hippocampus of lactating rats to damage induced by excitotoxic insults (Cabrera et al., 2013).

Progesterone and allopregnanolone attenuate traumatic brain injury, and diminish the elevation of pro-inflammatory cytokines in a time-dependent manner, suggesting that the protection occurs by limiting the overexpression of cytokines, when they peak at 3 h after a brain injury, rather than inhibiting their expression later in the post-injury cascade of toxic events (He et al., 2004). Both steroids prevent breakdown of the blood–brain barrier in edema and stroke (Ishrat et al., 2010). Progesterone exerts some of its actions through the intracellular, membrane-bound progesterone receptor, while allopregnanolone does not bind to the progesterone receptor (Ishrat et al., 2010). Also, progesterone can reduce the excessive excitotoxicity and inflammation by stimulating activation of the neuroprotective mitogen-activated protein kinase (MAPK) and PI3-K pathways (Kaur et al., 2007). Through use of calcium imaging, electrophysiology, and the measurement of changes in activity-dependent gene expression, progesterone was found to block calcium entry through voltage-gated calcium channels, leading to alterations in the signaling of the activity-dependent transcription factors nuclear factor of activated T-cells (NFAT) and cAMP response element-binding protein (CREB; Luoma et al., 2012). This effect of progesterone on calcium signaling provides a putative mechanism for its neuroprotective actions (Luoma et al., 2012). Furthermore, allopregnanolone is an allosteric modifier of the GABAA receptors expressed by oxytocin neurons (Koksma et al., 2003). Electrophysiological studies of hypothalamic oxytocin neurons showed that allopregnanolone acts via a G-protein mechanism involving protein kinase C to delay the closure of the Cl^-^ channel after activation (Brussaard and Koksma, 2003), enhancing both the tonic and phasic actions of GABA in oxytocin neurons. This modulatory action on GABA transmission represents an alternative candidate pathway for the protective actions of this steroid.

Progesterone also plays an important role in the periphery. Progesterone-dependent immunomodulation is one of the mechanisms that enable pregnancy to proceed to term, because it protects the fetus from immunological rejection. Recent evidence suggests that autocrine/paracrine factors such as cytokines play a crucial role, possible as effectors of steroid hormones (Agrawal et al., 2011). A growing body of evidence implicates progesterone in the establishment of an adequate immune response during pregnancy (Hirsch and Muhle, 2002; Aisemberg et al., 2013).

Thus, progesterone and its metabolite allopregnanolone are important modulators of the immune responses in the periphery and within the CNS. Such effects are aimed to diminish the impact of an acute lesion or degenerative process in the nervous system while in the periphery contributes to the regulation of immune response against the fetus (Aisemberg et al., 2013).

### PROLACTIN ACTIONS ON CNS AND IMMUNE SYSTEM

Prolactin is a peptide hormone secreted from the anterior pituitary into the circulation; it is thought to cross the blood–brain barrier and is known to regulate a wide variety of physiological process (Bole-feysot et al., 1998; Grattan and Kokay, 2008). PRL is also produced in a broad spectrum of extrapituitary sites including cells of the nervous and the immune system (Montgomery, 2001; Torner et al., 2002; Ignacak et al., 2012), and it is an important mediator of the immunoneuroendocrine network. However, effects of PRL on the immune system are complex. Removal of PRL by hypophysectomy impairs thymus growth (Nagy and Berczi, 1978) and immune reaction to immunogenic factors (Bernton et al., 1988) in rats. On the other hand, hyperprolactinemia (HP), in mice injected with *Listeria monocytogenes* increases mortality associated with impaired lymphocyte proliferation and decreased macrophage-activating factor production by T lymphocytes (Bernton et al., 1988). Furthermore, a PRL-like mRNA and a secreted product have been detected in human B-lymphoblastoid cell lines (Bernton et al., 1988; Baglia et al., 1991). Peripheral blood mononuclear cells also secrete a PRL-like protein, suggesting that it binds to PRL receptors and migrates to the nucleus, where it serves as a co-mitogen and autocrine regulator of cell growth. But, there are several reports showing that PRL is not essential for the proper development and function of the mouse immune system. Using PRL-deficient animals it was shown that PRL is not required for normal hematopoiesis (Horseman et al., 1997), and that PRL receptor signaling is not required for normal immunity (Bouchard et al., 1999). PRL is known to have other contradictory actions on the immune system that depends upon the concentration: it inhibits lymphocyte proliferation at high concentrations, while having enhancing effects at lower concentrations (Matera et al., 1992).

During pregnancy in rats, PRL plasma levels are high in the first half, they decrease until term, and then rise again at postpartum and throughout lactation (Neville et al., 2002). PRL actions in early gestation are crucial to prepare the mammary gland for lactation and in the CNS to establish the appropriate adaptive behavioral responses of the mother (Grattan and Kokay, 2008) toward the pups. PRL has been related to behavioral and neuronal effects, such as maternal behavior (Mann and Bridges, 2001), attenuation of anxiety and hormonal and neuronal responses to various stressors (Lightman et al., 2001; Torner and Neumann, 2002; Donner et al., 2007), neurogenesis (Shingo et al., 2003; Torner et al., 2009; Larsen and Grattan, 2010; Walker et al., 2012), and neuroprotection (Torner et al., 2009; Tejadilla et al., 2010).

Inflammatory response after a brain injury, such as proliferation and activation of glia is enhanced by PRL (Möderscheim et al., 2007), and elevated levels of PRL stimulate mitogenesis in astrocyte and oligodendrocyte populations of the subventricular zone (Larsen and Grattan, 2010). PRL also increases oligodendrocyte precursor cell proliferation, which in turn enhances the capacity to generate new oligodendrocytes and myelination. This process is associated with the capacity to repair white matter damage in the maternal CNS by increasing myelin basic protein expression (Gregg, 2009). During lactation, a physiological hyperprolactinemic state, there is a reduced sensitivity to kainic acid-induced cell damage in the dorsal hippocampus of the dam, showing that lactation is a natural model of neuroprotection (Vanoye-Carlo et al., 2008; Cabrera et al., 2009) in which PRL can participate. PRL systemic administration has been reported to protect the dorsal hippocampus of female rats against excitotoxicity induced by kainic acid administration, blocking cell loss and neurodegeneration, and diminishing the progression of kainate-generated behavioral manifestation of seizures (Tejadilla et al., 2010). Overall, these studies on protective effects of PRL within the CNS draw attention to the importance of studying females, and the relevance of neuroendocrine–immune interactions when investigating effects of this hormone.

An interesting example of the neuroendocrine–immune interactions is seen in the HP occurring in patients with autoimmune diseases, such as rheumatoid arthritis and systemic lupus erythematosus, and with organ-specific autoimmune diseases, as celiac disease, type 1 diabetes mellitus, Addison’s disease, autoimmune thyroid diseases (reviewed by, De Bellis et al., 2005). In these diseases PRL increases the synthesis of IFNγ and IL-2 by Th1 lymphocytes. Moreover, PRL activates Th2 lymphocytes with autoantibody production. The inhibitory effects of IL-1ß on the tuberoinfundibular dopaminergic neurons that inhibit PRL secretion could explain this HP. González et al. (2004), showed that i.c.v. injection of LPS in rats, produces a decrease in tyrosine hydroxylase (TH) activity in the medial eminence, an increase in the serum levels of PRL, and a decrease in the number of TH- and TH mRNA-positive cells in the arcuate nucleus, indicating that dopamine neurons of the hypothalamus are functionally susceptible to local inflammatory stimuli. Additionally, treatment with the dopamine-agonist, bromocriptine, inhibits both PRL secretion and the severity of acute experimental encephalomyelitis (Riskind et al., 1991). This is an example of how molecules of the immune system could affect the neurons in the hypothalamus increasing the secretion of PRL, which in its turn will enhance peripheral inflammatory responses.

## CONCLUSION

In summary, during pregnancy and lactation, responses of the HPA axis and the immune system are altered and clearly regulated by suckling and hormone fluctuation. Pregnancy and lactation are the most important periods for the conservation of the species, and they represent fundamental stages at which both mother and offspring must be protected. The immune system is crucial to protect the mother and the product against the environment and there is evidence supporting the notion that immunity is suppressed during motherhood. In this sense, the immune responses of the mother should be adjusted to conserve defenses, but should be tuned to preserve the developing offspring (Mor and Cardenas, 2010). Therefore, pregnancy and lactation are unique conditions in which the immune system is modulated or adjusted, but not fully suppressed.

## AUTHOR CONTRIBUTIONS

Nela Monasterio conceived the main premises that are the focus of this review and wrote the largest portion of the paper; Edgar Vergara wrote important subsections of the paper; and Teresa Morales conceived the main premises that are the focus of this review, designed the structure of the paper, wrote a large portion of the paper, and edited the final version.

## Conflict of Interest Statement

The authors declare that the research was conducted in the absence of any commercial or financial relationships that could be construed as a potential conflict of interest.
